# Differential roles of polar orbital prefrontal cortex and parietal lobes in logical reasoning with neutral and negative emotional content

**DOI:** 10.1016/j.neuropsychologia.2018.05.014

**Published:** 2018-10

**Authors:** Iveta Eimontaite, Vinod Goel, Vanessa Raymont, Frank Krueger, Igor Schindler, Jordan Grafman

**Affiliations:** a Department of Psychology, University of Hull, UK; b Department of Psychology, York University, 4700 Keele St., Toronto, Ont., Canada M3J 1P3; c Department of Radiology, Johns Hopkins University, Baltimore, MD, USA; d Department of Medicine, Imperial College, London, UK; e School of Systems Biology, George Mason University, Fairfax, VA, USA; f Department of Psychology, George Mason University, Fairfax, VA, USA; g Northwestern University Medical School, Cognitive Neurology and Psychiatry and Behavioral Sciences and Physical Medicine and Rehabilitation, Chicago, IL, USA

**Keywords:** Reasoning, Emotional content, Neutral content, Exclusive disjunctions, Polar/orbital PFC, Parietal lobe, Penetrating traumatic brain injury, Logic, Rationality

## Abstract

To answer the question of how brain pathology affects reasoning about negative emotional content, we administered a disjunctive logical reasoning task involving arguments with neutral content (e.g. Either there are tigers or women in NYC, but not both; There are no tigers in NYC; There are women in NYC) and emotionally laden content (e.g. Either there are pedophiles or politicians in Texas, but not both; There are politicians in Texas; There are no pedophiles in Texas) to 92 neurological patients with focal lesions to various parts of the brain. A Voxel Lesion Symptom Mapping (VLSM) analysis identified 16 patients, all with lesions to the orbital polar prefrontal cortex (BA 10 & 11), as being selectively impaired in the emotional reasoning condition. Another 17 patients, all with lesions to the parietal cortex, were identified as being impaired in the neutral content condition. The reasoning scores of these two patient groups, along with 23 matched normal controls, underwent additional analysis to explore the effect of belief bias. This analysis revealed that the differences identified above were largely driven by trials where there was an incongruency between the believability of the conclusion and the validity of the argument (i.e. valid argument/false conclusion or invalid argument/true conclusion). Patients with lesions to polar orbital prefrontal cortex underperformed in incongruent emotional content trials and over performed in incongruent neutral content trials (compared to both normal controls and patients with parietal lobe lesions). Patients with lesions to parietal lobes underperformed normal controls (at a trend level) in neutral trials where there was a congruency between the believability of the conclusion and the validity of the argument (i.e. valid argument/true conclusion or invalid argument/false conclusion). We conclude that lesions to the polar orbital prefrontal cortex (i) prevent these patients from enjoying any emotionally induced cognitive boost, and (ii) block the belief bias processing route in the neutral condition. Lesions to parietal lobes result in a generalized impairment in logical reasoning with neutral content.

## Highlights

• Polar/orbital PFC lesions result in impaired reasoning in emotional content reasoning.
• Polar/orbital PFC lesion patients overperform in neutral content reasoning trials.
• These differences were driven by incongruent reasoning trials.
• Parietal lesions result in impaired reasoning with neutral but not emotional content.

## 1 Introduction

Everyday life is affected by our ability to reason; and although the majority of choices are not structured as problems of formal reasoning, humans still have to apply principles of rationality to determine which choices will lead to the desired outcomes. Evidence from experimental investigations into the reasoning process, however, suggests that a view of reasoning as a purely logical process is too simplistic: real-world reasoning depends on various factors such as conclusion believability (Evans, 2003, Evans et al., 1983) and emotional content (Blanchette and Caparos, 2013, Goel and Vartanian, 2011, Goel et al., 2017) of arguments.

While the issue of belief bias has long been recognized in the psychological literature on reasoning (Evans et al., 1983, Wilkins, 1929), the effect of emotional content has received much less attention (Blanchette and Richards, 2004, Blanchette, 2006, Goel and Vartanian, 2011). Even within the cognitive neuroscience literature, while a number of studies have explored the effects of belief bias (Goel et al., 2000, Goel and Dolan, 2003a, Tsujii et al., 2010, Tsujii and Watanabe, 2009), studies of the effect of emotions on logical reasoning are rarer (e.g. Goel et al., 2017; Goel and Dolan, 2003b). However, in the decision making literature the relationship between emotion and cognition, has been explored more widely (e.g. Andrade and Ariely, 2009; Bechara and Damasio, 2005; Hu et al., 2015).

In an early neuroimaging study, Goel and Dolan (2003b) observed that while the left dorsolateral prefrontal cortex (BA 44, 8) was activated during neutral reasoning trials, reasoning with emotionally charged syllogisms activated bilateral ventromedial prefrontal cortex (vmPFC; BA 11/25). Consistent with this finding, lesion studies have shown that neurological patients with lesions to left lateral and superior medial frontal areas are impaired on simple deductive reasoning problems (Reverberi et al., 2009). More recently it has been shown that patients with focal lesions to polar/orbital prefrontal cortex (BA 10 and 11) are selectively impaired in reasoning with categorical syllogisms with emotional content, but not on the same syllogistic forms involving neutral content (Goel et al., 2017).

The current study builds upon and extends Goel et al. (2017) along task and methodological lines. In terms of task, we address whether the results generalize to logical forms beyond categorical syllogisms. In terms of methodology, rather than pre-selecting patients with lesions in polar/orbital prefrontal cortex (BA 10 and 11), we subjected 92 patients with lesions extending to various parts of the brain, to a voxel-based lesion-symptom mapping (VLSM) analysis. This allows us to explicitly test whether lesions in regions beyond anterior/orbital PFC affect reasoning about emotional content.

With respect to logical forms, Goel et al. (2017), following up on Goel and Dolan, 2003a, Goel and Dolan, 2003b, utilized categorical syllogism tasks, as have many studies of reasoning (Blanchette and Campbell, 2012, Bonnefond and Van der Henst, 2009, Goel et al., 2017, Goel et al., 2000, Goel and Vartanian, 2011). Categorical syllogisms test our understanding of quantification and negation (e.g All A are B; All B are C; No A are C). In the current study we chose to analyse arguments containing disjunctive operators, such as the following:

A or B but not both/Not A/Therefore B

These arguments test our logical understanding of the ‘exclusive or’ operator (Hurford, 1974, Newstead et al., 1984, Noveck et al., 2002, Reverberi et al., 2007).

The issue of the specific nature of the reasoning task is important. Reasoning is often thought of as a unitary phenomenon, but 20 years of neuroimaging research on the topic reveals that different logical argument forms recruit different neural machinery (Goel, 2007, Goel et al., 1997, Prado et al., 2011) So, an outstanding question is whether the role of inferior/orbital PFC in reasoning with emotional content articulated in Goel et al. (2017) is specific to categorical syllogisms or generalizes beyond them, perhaps to all logical reasoning forms.

Given that Goel et al. (2017) postulated a reasoning independent role for polar orbital PFC, we hypothesized that patients with lesions to the polar and orbital cortex (mainly BA 10 and BA 11) would be selectively impaired in disjunctive reasoning *with emotional content* but not with neutral content.

## 2 Methods

### 2.1 Participants

**Fig. 1:**
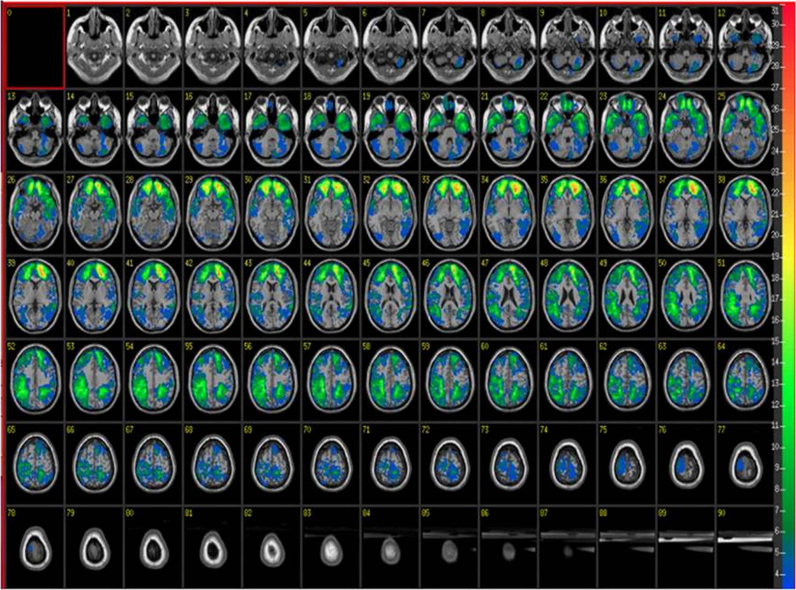
Lesion overlay of all 92 patients. The colour bar indicates the number of patients with lesion in that area. Images are shown following radiological convention (Left = Right, Right = Left).

Participants were drawn from the Phase III of the Vietnam Head Injury Study (VHIS) registry. This registry contains data from Vietnam war veterans who served in Vietnam in the late 1960's and early 1970's (Barbey et al., 2012, Grafman et al., 1988, Koenigs et al., 2009, Raymont et al., 2010). Ninety-two Vietnam War veterans with focal brain lesions due to penetrating shrapnel wounds completed the task. Twenty-three Vietnam War veterans, with combat experience, but without brain injury, also completed the task and served as normal controls. The two groups were matched in terms of age, education, and cognitive assessment scores (see Section 2.3). The lesion volumes of the patient group ranged from 1 cc to 97.13 cc, but were not larger than 75 cc on a single hemisphere. Thirty-eight patients had unilateral focal lesions to the right hemisphere, 31 had unilateral focal lesions to the left hemisphere, and 23 patients had bilateral focal lesions. The location of lesions varied between patients, although the majority showed prefrontal, temporal or parietal damage. It should be noted that not all brain areas were affected by lesions, in particular, left lateral PFC (BA 44, 45) was largely spared in this cohort of patients. A lesion overlay map is provided in Fig. 1. The language, motor and sensory functions of the patients were sufficiently intact to take part in the experiment (see also Section 2.3 for details).

### 2.2 Lesion assessment

Patients CT axial scans without contrasts were acquired at Bethesda Naval Hospital on a GE Medical Systems Light Speed Plus CT scanner in helical mode (150 slices per subject, field of view covering head only). Images were reconstructed with an in-plane voxel size of .4 × .4 mm, overlapping slice thickness of 2.5 mm, and a 1 mm slice interval. A trained neuropsychiatrist (V.R.) with clinical experience in neuropsychological testing manually traced lesions in all relevant slices of the CT scan images in native space. Following this another investigator (J.G.), blind to the results of the psychological testing, reviewed the lesions for consensus.

Spatial normalization to a CT template brain in MNI space was achieved stepwise: first, all non-brain tissue was automatically removed using the BET algorithm in MEDx (Medical Numerics). Following this, the automated image registration (AIR) algorithm (Woods et al., 1993) using a 12-parameter affine linear transformation was applied to individual brain volumes allowing for translation, rotation, scaling and shearing to normalize the volume to a reference template volume of an MRI of a 27-year-old normal male, which was later transformed to Talairach space using a 12-parameter affine linear transformation. The lesion voxels in the normalization process were not included. Lesion location and volume were determined from CT images using the Analysis of Brain Lesion software (ABLe; Makale et al., 2002; Solomon et al., 2007) contained in MEDx v3.44 (Medical Numerics) with enhancements to support the Automated Anatomical Labelling atlas (Tzourio-Mazoyer et al., 2002). The resulting normalised lesion mask was used in the VLSM analysis for each subject.

### 2.3 Cognitive assessment

To assess the patients’ cognitive, emotional and psychological functioning, an extensive battery of tests was administered to all participants. The ones deemed most relevant for our purposes are the Wechsler Adult Intelligence Scale (WAIS-III; Wechsler, 2008)), the Wechsler Memory Scale (WMS-III; Wechsler, 1997)) the Beck's Depression Inventory (BDI) and the Structural Clinical Interview for DSM-IV (SCID). These results are reported in Table 1. In addition, pre-injury intelligence was assessed with the Armed Forces Qualification Test (AFQT-7A) taken by each (non-officer) participant upon their entry into the military. This test has been standardised within the U.S. military and the scores are found to be highly positively correlated with WAIS IQ scores (Grafman et al., 1988). The scores from this test are given as percentiles (Table 1). All the patients were right-handed males aged between 52 and 70 years with similar years of formal education (Table 1).

**Table 1:** Demographic and assessment data for patients and normal controls. Mean percentiles (SD) are provided for Pre-injury IQ (AFQT-7A) and means for the rest of the results.

	Patients	Normal controls
N	92	23
Age	58.17 (2.51)	58.69 (2.96)
Education (years)	14.91 (2.31)	14.23 (1.97)
Verbal IQ (WAIS)	106.16 (13.34)	104.61 (10.17)
Performance IQ (WAIS)	101.13 (14.81)	106.13 (11.41)
Full Scale IQ (WAIS)	104.59 (13.31)	105.61 (8.39)
Working Memory (WMS)	101.17 (12.92)	104.57 (13.06)
Pre-injury IQ (AFQ T-7A)	61.47 (25.24)	62.05 (21.04)
BDI	8.47 (8.60)	10.22 (8.96)
SCID	75.87 (13.73)	73.87 (11.38)

To investigate whether there were any significant differences in the cognitive baseline evaluation between the 92 patients and normal controls, independent *t*-test were performed on the above mentioned measures. Only years of education showed a trend level advantage for patients compared to normal controls (Mean = 15.11, SD = 2.23 vs. Mean = 14.23, SD = 1.97), but this difference was not significant (*t*(108) = 1.69, *p* = .093, 95% CI [− 0.06, 1.82], Cohen's *d*_*s*_ = .322). Other measures did not reveal significant differences between the 92 patients and 23 normal controls (*t*(112) ≤ 1.39, *p* ≥ .167, Cohen's *d*_*s*_ = .260).

### 2.4 Task and procedure

Participants were presented with 8 exclusive disjunctive arguments distributed among 64 arguments of other argument types including, implication, conjunction, and categorical syllogisms. Half of each type of argument contained emotional negative content, and other half neutral content. Exclusive disjunctions were constructed along the following lines:

Premise 1: “Either there exist pink elephants or white mice, but not both.”

Premise 2: “There are no white mice.”

Conclusion: “There are pink elephants.”

The disjunctions contained 4 emotional trials, and 4 neutral trials. All emotional content was negatively valanced. Examples of emotional and neutral exclusive disjunctions can be found in Table 2 (the complete list of items is reproduced in the Supplementary material).

**Table 2:** Examples of emotional and neutral exclusive disjunctions by content.

	Emotional disjunction	Neutral disjunction
Congruent	Either in Korea there are edible dogs or opossums, but not both.	Either there are Christians or atheists in America, but not both.
	In Korea there are no opossums.	There are no Christians in America.
	In Korea there are edible dogs.	There are no atheists in America.
	
Incongruent	Either there are paedophiles or politicians in Texas, but not both.	Either there exist pink elephants or white mice, but not both.
	There are politicians in Texas.	There are no white mice.
	There are no paedophiles in Texas.	There are pink elephants.

One of the most robust findings in the psychology of reasoning literature is the belief bias effect (Evans et al., 1983, Wilkins, 1929). It is the finding that the believability of the conclusion affects reasoning accuracy. Arguments where the validity judgment and conclusion believability go in the same direction (valid/believable and invalid/unbelievable) are considered to be congruent (see Table 2). Arguments where the validity judgment and the conclusion believability contradict (invalid/believable and valid/unbelievable) are referred to as incongruent (see Table 2). Accuracy rates for congruent arguments are much higher than accuracy rates for incongruent arguments (Evans et al., 1983). Therefore the arguments were counterbalanced to include an even number of congruent (believable/valid and unbelievable/invalid) and incongruent items (unbelievable/valid and believable/invalid). As a result, there were two congruent emotional trials, two incongruent emotional trials, two congruent neutral trials and two incongruent neutral trials. The selected valid or unbelievable presentation order of the neutral and emotional disjunctions was counterbalanced between-subjects, and the order of disjunctions within neutral or emotional content was randomised. The stimuli were presented on a computer screen (pixel resolution 512 × 384) with SuperLab v1.5 Software.

The concept of validity and the task was explained to participants both conceptually and with concrete examples. The explanation and examples continued until the experimenter was satisfied that the participant understood the notion of validity and the task. Task instructions then appeared in writing.

The experiment had three parts. In the first part, participants were shown two premises and a conclusion and asked to determine if the latter followed from the former. If it did, participants had to press the designated “yes” key, and if not, they had to press the designated “no” key. Each trial remained on screen until participants responded. Their response triggered the next trial. Participants were encouraged to be as accurate and as quick as possible, but there were no time constraints. All participants completed all the trials. After completion of the reasoning task, participants were given a break with no time constrains.

In the second part of the experiment, the conclusions (3rd sentence) of the logical arguments from the first part were presented on the screen, and the participants were asked to rate the believability of the sentence on the scale from 1 to 5, where 1-represented very unbelievable and 5-represented very believable. These ratings were used to control for the belief bias effect (see Section 2.5). Another rest period followed part two.

In the third part, the conclusions of the arguments were re-presented and the participants were asked to judge each conclusion on its emotional arousal on the scale from 1 to 4 (1 – negative emotion/high arousal, 2 – negative emotion/low arousal, 3 – positive emotion/high arousal, 4 – positive emotion/low arousal).

### 2.5 Belief bias reclassification

Given the importance of the belief bias effect, we counterbalanced the reasoning material for congruent and incongruent trials, as noted above. However, this balancing is only effective insofar as participants share our beliefs about the conclusions. To overcome this issue the reasoning items were re-classified after the experiment based on each participants’ actual belief rating for each of the conclusions. This was done to ensure that the congruency and incongruency classifications were made based on the participants’ actual beliefs, rather than our beliefs.

For all trials where participants indicated a belief rating of 3, the initial congruency classification was maintained, but the invalid trials which participants indicated to be unbelievable (ratings of 1 and 2) and valid trials that were rated believable (4 and 5) were classified as congruent. Conversely, ratings of 1 and 2 for valid trials and ratings of 4 and 5 for invalid trials were classified as incongruent. The average number of congruent and incongruent trials remained relatively balanced after the adjustment (i.e., neutral congruent vs. neutral incongruent = 1.25 (0.46) vs. 2.75 (0.46); emotional congruent vs. emotional incongruent = 1.96 (0.46) vs. 2.03 (0.46).)

### 2.6 Data analysis

#### 2.6.1 VLSM

We began by performing a VLSM analysis, using non-parametric Mann-Whitney *U* test, (with automated anatomical labelling (AAL)) in ABLe on all patients (Solomon et al., 2007). VLSM analyses associate behaviour with lesion site, on a voxel-by-voxel basis, by grouping patients into those who show a lesion in a particular voxel and comparing their behavioural task performance with that of all other patients (Solomon et al., 2007). Non-parametric tests were used due to the small number of trials in each task condition – only 4 trials in emotional and 4 in neutral content – and an abnormal distribution of behavioural scores according to Kolmogorov-Smirnov and Shapiro-Wilk tests (as has been done previously by e.g. Brosig, 2002; Falk et al., 2005). For the current study, three behavioural measures were entered into the analysis: mean correct responses for all exclusive disjunctions, mean correct responses for emotional trials, and mean correct responses for neutral trials.

A false-discovery rate (FDR) correction of .05 was applied for multiple comparisons. The minimum cluster size was set to 10 voxels and only those voxels where at least three patients showed overlapping lesions were used for the analysis.

#### 2.6.2 ANOVA

As VLSM analysis in ABLe only allows for simple *t*-tests, we undertook a second level analysis in which we incorporated the normal controls and broke down the behavioural scores to include the belief bias effect, resulting in a Group (PFC, parietal, NC) by Content (Neutral, Emotional) by Congruency (Congruent, Incongruent) mixed design. We used the R-package for nonparametric ANOVA-type statistics analysis (nparLD function, Noguchi et al., 2012) and further analysis of the interactions was explored with a non-parametric mixed-design Friedman's ANOVA (Field et al., 2012). The dependent variable was correct response to the reasoning task in the various conditions. Significant main effects and interactions were followed-up by non-parametric *post hoc* tests; between-subject differences were explored with planned Mann-Whitney *U* tests adjusted for multiple comparisons (Bonferroni-corrected, alpha = 0.017, two-tailed). Wilcoxon Signed-Rank tests were used for evaluating within-subject differences. Given the small number of stimuli per category, exact p-values (Monte Carlo simulation) for each post hoc test were computed and are provided in addition to the asymptotic p-values (Mehta and Patel, 2011). Nonparametric test effect sizes were measured as suggested by Tomczak and Tomczak (2014) and parametric tests effect sized were calculated according to Lakens (2013). Non-significant results were summarised by reporting the closest one to significance, for example: *t*(31) = 1.98, *p* = .057, Cohen's *d*_*s*_ = .69, and *t*(31) = 0.98, *p* = .335, Cohen's *d*_*s*_ = .34, would be reported as *t*(31) ≤ 1.98, *p* ≥ .057, Cohen's *d*_*s*_ ≤ .69. Finally, to ensure that our patient results were not driven by a few individuals, we checked for outliers in our three groups (PFC patients, parietal lobe patients, normal controls). No outliers were found.

## 3 Results

### 3.1 VLSM patients only analysis

**Fig. 2:**
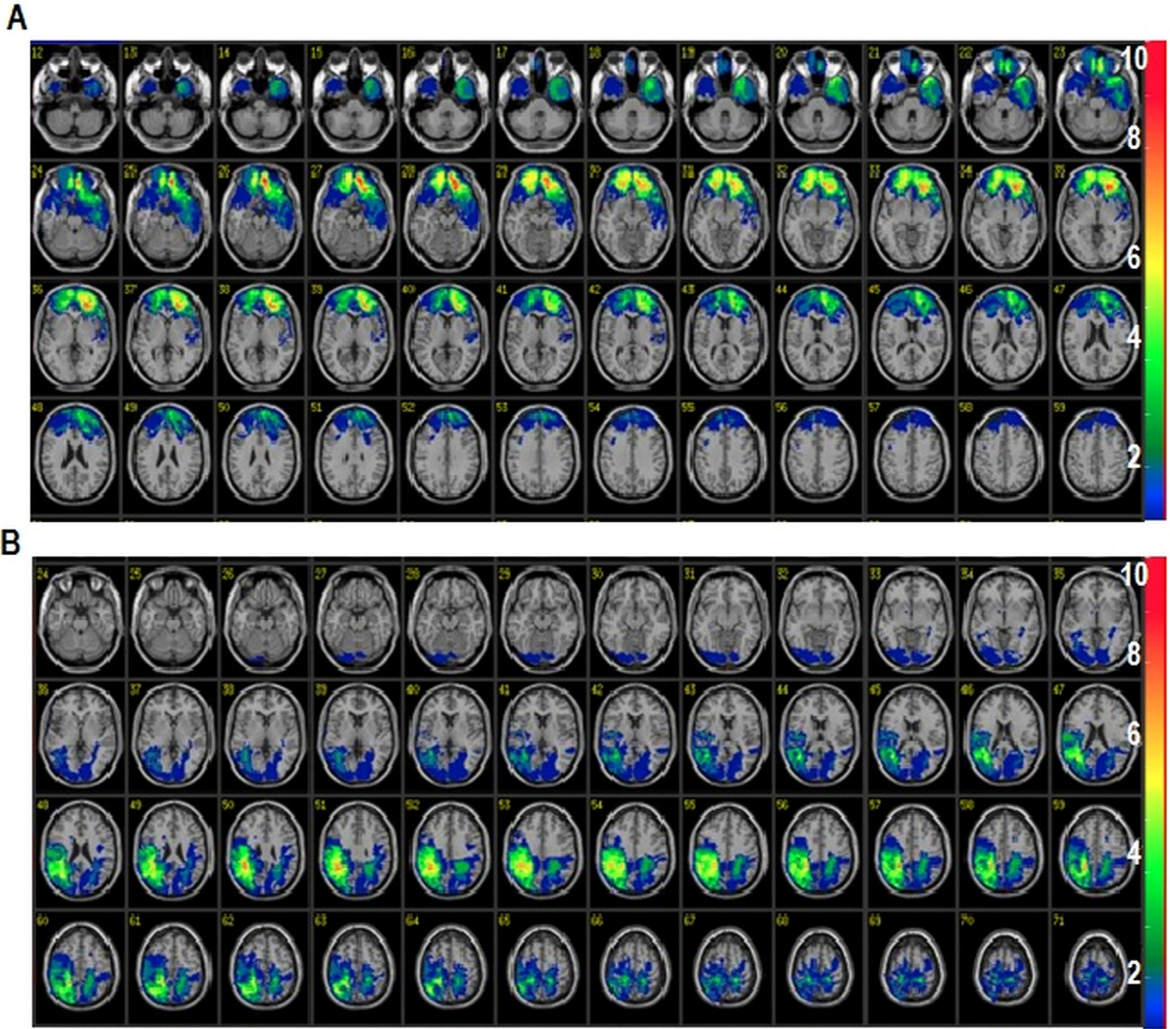
Lesion overlay maps for patients displayed on the VOTL Atlas found in ABLe software with overlap threshold of 2. (A) Slices 12 – 59 of polar/orbital PFC lesion patients group (n = 16); (B) Slices 24 – 71 of parietal lesion patients group (n = 17). Images are presented in radiological convention (Left = Right, Right = Left).

The VLSM analysis with automated anatomical labelling (AAL) indicated significant results for the emotional content trials. Analysis revealed that only patients having lesions mainly to the left and right frontal lobes (Talairach coordinates [− 16, 40, − 16], [16, 38, − 22], and [8, 58, − 6], N = 16; Fig. 2A and Table S1) performed poorly in emotional exclusive disjunctions compared to other patients (Table 4). As indicated in Fig. 2A and Table S2 (Supplementary material), all of these 16 patients have lesions largely confined to BA 10 and BA 11.

**Fig. 3:**
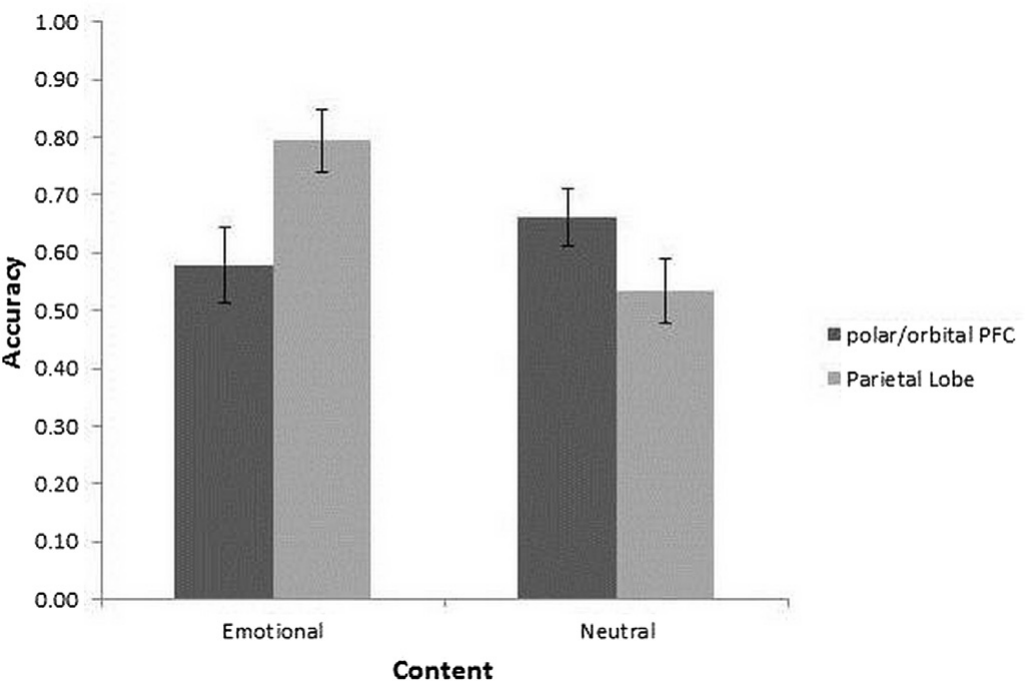
Mean accuracy rates (± 1 SEM) for the groups determined by VLSM analysis as a function of argument content.

VLSM analysis also identified another patient group of 17 patients who were impaired on neutral exclusive disjunctions compared other patients (Table 4). As noted in Fig. 2B and Table S2, these patients had lesions largely confined to right parietal areas (BA 7 & 40, Talairach coordinates [46, − 50, 28] and [24, − 62, 32], N = 17). These results indicate a double disassociation between the two patient groups as determined by the VLSM analysis (Fig. 3).

The cognitive baseline scores and volume loss data for these two patient groups (and normal controls)^1^ is presented in Table 3. To control for possible confounding variables, potential differences between the two patient groups (polar/orbital PFC, parietal lobe) with respect to education, BDI, SCID, pre-Injury IQ, WAIS Verbal IQ, Performance IQ and Full IQ, WMS Working Memory Primary Index Scores, and overall lesion volumes (cubic centimetres) were tested with an independent *t*-test for each measure separately. T-test results showed no significant difference between the two patient groups in all these measures (*t*(31) ≤ 1.98, *p* ≥ .057, Cohen's *d*_*s*_ ≤ .69; Table 3).

**Table 3:** Clinical and demographic information (SD) from polar/orbital (PFC), parietal lobe (PL) and normal controls (NC) samples.

	PFC	PL	NC
Number of Patients	16	17	23
Age (years)	57.50 (1.15)	59.00 (3.10)	58.69 (2.96)
Education (years)	14.13 (2.53)	15.56 (2.12)	14.22 (1.97)
Pre-injury IQ (AFQ T-7A)	57.31 (26.01)	62.13 (26.65)	62.05 (21.04)
WAIS			
Verbal IQ	102.63 (7.61)	108.76 (9.98)	104.61 (10.17)
Performance IQ	103.60 (9.91)	101.41 (14.94)	106.13 (11.41)
Full Scale IQ	104.00 (6.14)	106.76 (12.09)	105.61 (8.39)
WMS Working Memory Primary Index Score	100.20 (11.69)	103.41 (12.56)	104.56 (13.05)
BDI	8.69 (8.28)	7.00 (6.18)	10.22 (8.96)
SCID	79.44 (11.99)	78.41 (9.08)	73.87 (11.38)
Total Volume Loss (cc)	41.15 (26.49)	38.47 (27.69)	0

WAIS = Wechsler Adult Intelligence Scale; WMS = Wechsler Memory Scale; BDI = Beck Depression Inventory; SCID-GAF = Structural Clinical Interview for DSM-IV – Global Assessment Function; PFC = Polar/Orbital Prefrontal Cortex; PL = Parietal Lobe; NC = Normal Controls.

We also compared the cognitive baseline scores of normal controls and the two patient groups (polar/orbital PFC, parietal lobe) with one-way ANOVAs, for each measure separately. ANOVA results showed no significant difference between the three participant groups in any of these measures (*F*(2, 55) ≤ 2.55, *p* ≥ .088, η_p_^2^ ≤ .08; Table 3).

### 3.2 ANOVA including normal controls and belief bias

**Fig. 4:**
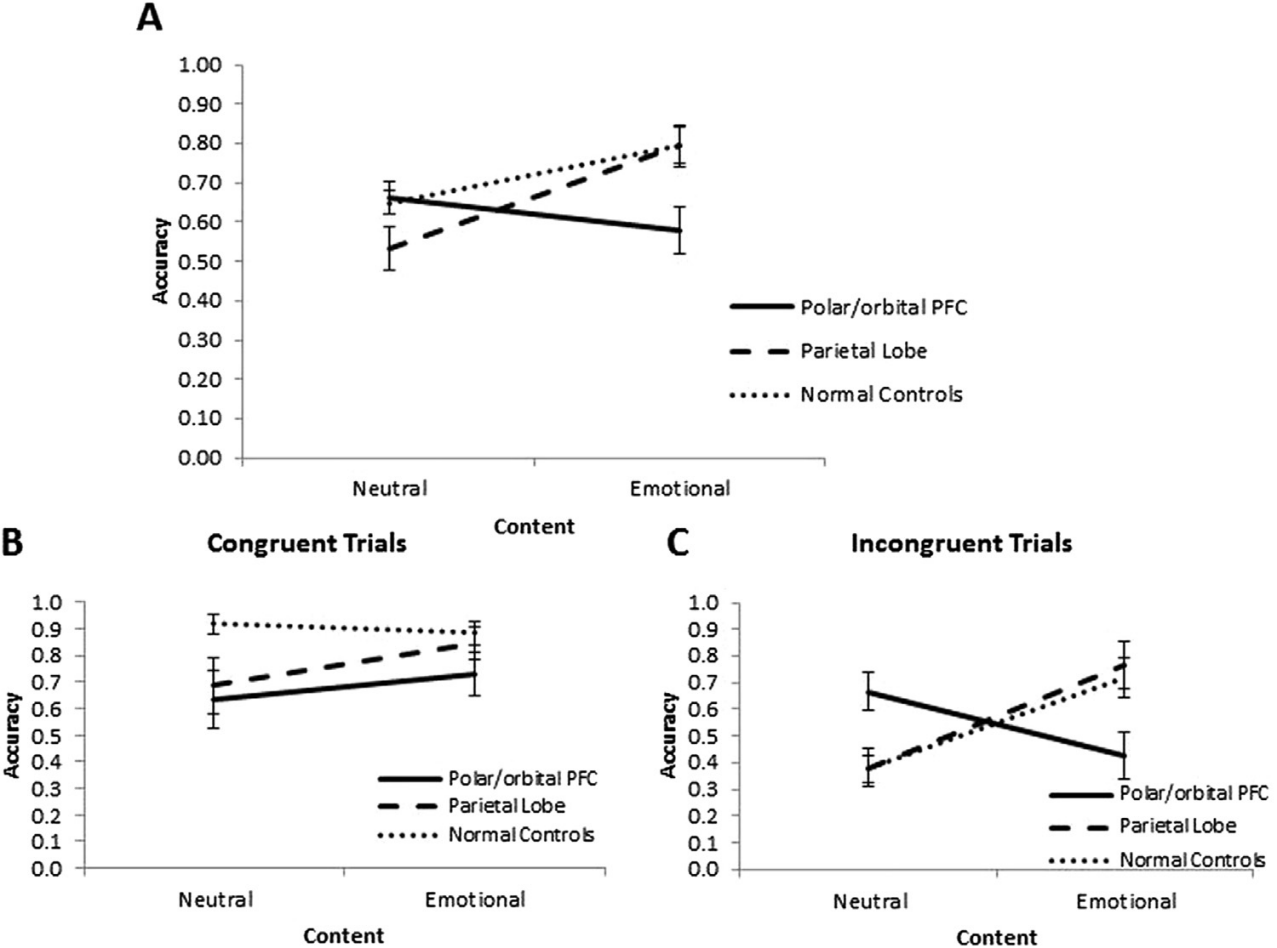
Mean accuracy rates (± 1 SEM) for (A) Exclusive Disjunction items, (B) Exclusive Congruent Disjunction items and (C) Exclusive Incongruent Disjunction items as a function of Content (Emotional, Neutral) and group (polar/orbital (PFC), parietal lobe (PL), normal controls (NC)).

To compare the performance of patients with normal controls, and to explore the effect of belief bias, we added normal controls to Group, separated the reasoning trials into Congruent and Incongruent items, and carried out nonparametric ANOVA-type statistics (ATS) with between subject factor Group (NC, polar/orbital PFC, PL) and within-subject factors Content (Emotional and Neutral) and Congruency (Congruent and Incongruent) and a dependent variable of accuracy. The results with non-parametric ANOVA-type statistics indicated the main effects of Congruency and of Content to be significant (*ATS*(1) = 22.61, *p* ≤ .001, and *ATS*(1) = 9.95, *p* = .002, respectively). The Group by Content interaction was also significant (*ATS*(1.94) = 7.07, *p* = .001) as well as three-way Group by Content by Congruency interaction (*ATS*(1.67) = 5.68, *p* = .006). The main effect of Group, and Congruency by Content, and Group by Congruency interactions were not significant (*ATS*(1.93) = 2.35, *p* = .11, *ATS*(1) = 2.61, *p* = .11, and *ATS*(1.87) = 1.57, *p* = .21, respectively). The accuracy scores for each condition are reproduced in Table 4 and Fig. 4. These scores are consistent with those reported in the literature, on similar disjunctive reasoning tasks with normal, healthy, university student participants (García-Madruga et al., 2001; Reverberi et al., 2007).

**Table 4:** Mean (SD) accuracy for emotional and neutral content overall, congruent and incongruent trials for polar/orbital PFC (PFC), parietal lobe (PL) and normal controls (NC) samples.

	Emotional			Neutral		
	Congruent	Incongruent	Overall	Congruent	Incongruent	Overall
polar/orbital PFC	0.73 (0.34)	0.43 (0.36)	0.58 (0.24)	0.64 (0.43)	0.67 (0.27)	0.66 (0.14)
PL	0.84 (0.25)	0.76 (0.36)	0.79 (0.22)	0.69 (0.43)	0.38 (0.30)	0.53 (0.23)
NC	0.88 (0.20)	0.72 (0.37)	0.79 (0.22)	0.92 (0.18)	0.38 (0.25)	0.65 (0.15)

To explore the significant three way interaction, reasoning trials were separated into Congruent and Incongruent and analysed separately.

#### 3.2.1 Congruent trials

A 2 × 3 Friedman's non-parametric mixed design ANOVA with a within-subject factor Content (Emotional and Neutral) and a between-subject factor Group (NC, polar/orbital PFC, PL) for Congruent items indicated no significant main effects of Group (*Q* = 2.50, *p* = .121) and Content (*Q* = 0.39, *p* = .542), as well as no significant Group by Content interaction (*Q* = 0.60, *p* = .564; Fig. 4).^2^

#### 3.2.2 Incongruent trials

Friedman's ANOVA for the Incongruent trials revealed significant main effect of Content (*Q =* 9.44, *p* = .005) as well as a significant Content by Group interaction (*Q* = 10.93, *p* ≤ .001; Fig. 4), but the main effect of Group was not significant (*Q =* 0.52, *p* = .604). *Post-hoc* comparisons for Emotional Content accuracy rates showed a significantly lower accuracy for polar/orbital PFC vs. PL (*U* = 69.50, *p* = .011, exact *p* = .010, *r* = .441) and a trend significance vs. NC (*U* = 106.00, *p* = .019, exact *p* = .020, *r* = .375, Bonferroni corrected two-tailed alpha < .017; Table 4). There was no significant difference between PL and NC (*U* = 180.50, *p* = .640, exact *p* = .682, *r* = .074). By contrast, in the Neutral content condition there was a significantly higher accuracy for polar/orbital PFC patients vs. PL patients (*U* = 70.00, *p* = .014, exact *p* = .013, *r* = .426) and vs. NC (*U* = 91.50, *p* = .006, exact *p* = .006, *r* = .437), but no significant difference between PL patients and NC (*U* = 193.50, *p* = .955, exact *p* = .976, *r* = .009).

The within group comparisons between Emotional Incongruent and Neutral Incongruent trials revealed a significant increase in accuracy rates from Neutral to Emotional trials for the NC group (*Z* = − 3.12, *p* = .002, exact *p* = .001, *r* = .651) and for the PL group (*Z* = − 2.57, *p* = .010, exact *p* = .008, *r* = .623). The decrease in accuracy from Neutral to Emotional trials for the polar/orbital PFC group was not significant (*Z* = 1.83, *p* = .067, exact *p* = .073, *r* = .458).

The results split by argument validity (valid and invalid) are presented in Supplementary Fig. S1. The figure confirms that results are not driven by a bias to reject arguments when the content is emotionally negative.

### 3.3 Response times

In order to evaluate if negative emotional content encouraged longer deliberations, a nonparametric ANOVA-type statistics (ATS) with between subject factor Group (NC, polar/orbital PFC, PL) and within-subject factors Content (Emotional and Neutral) and Congruency (Congruent and Incongruent) and a dependent variable of response time for accurate trials was carried out. However, the analysis indicated no significant main effects or interactions (*ATS*(1) ≤ 2.93, *p* ≥ .086).

### 3.4 Belief strength and emotional arousal

The possibility that the polar/orbital PFC groups’ impaired performance on the Emotional trials might be due to stronger (or weaker) participants’ belief or emotional arousal by the reasoning items was explored further.

#### 3.4.1 Belief strength

At first the mean belief rating for all disjunctions was calculated separately for each participant and then compared between groups. The between group analysis showed that there was no significant difference in belief strength between the groups (χ^2^ (2) = 0.558, *p* = .757, exact *p* = .761, Cramer's *V* = .075). Subsequently, belief ratings for conclusions intended to be true and conclusions intended to be false were also compared. Analysis did not identify any significant differences between the groups (true conclusion: χ^2^ (2) = 0.949, *p* = .622, exact *p* = .626, Cramer's *V* = .098; false conclusion: χ^2^ (2) = 0.198, *p* = .906, exact *p* = .905, Cramer's *V* = .045). Finally, the comparison of belief strength between Congruent and Incongruent trials in Emotional and Neutral trials did not show any significant differences between groups (χ^2^ (2) ≤ 2.23, *p* ≥ .328, exact *p* ≥ .334, Cramer's *V* ≤ .141).

#### 3.4.2 Emotional arousal

For each valance/arousal category (negative aroused, positive aroused, negative calm, positive calm) the proportion of the number of emotional trials rated in a specific category to the number of related emotional trials was calculated for each participant for all Emotional trials and for Congruent and Incongruent Emotional trials (as done by Goel et al., 2017). For example, if participants provided rating for all 4 trials, and 1 trial was “negatively calm”, the proportion of these type of trials would be 1 out of 4, or .25. Following this, the group differences in the mean proportion of ratings of each valance/arousal categories were investigated. The mean proportion rating for each valance/arousal category did not reveal any significant differences between groups in overall Emotional trials (χ^2^ (2) ≤ 2.27, *p* ≥ .321, exact *p* ≥ .339, Cramer's *V* ≤ .152). Emotional Incongruent and Emotional Congruent trials showed no significant difference between the groups in negative aroused, positive aroused, negative calm, positive calm categories (χ^2^ (2) ≤ 4.41, *p* ≥ .110, exact *p* ≥ .114, Cramer's *V* ≤ .212).

Taken together, there was no significant difference in belief strength or emotional arousal between the groups that could have affected their performance.

### 3.5 Correlational analysis

To investigate the associations between performance on Emotional and Neutral content trials (overall and separated by congruency) and percentage volume loss (collapsed across hemispheres), non-parametric Spearman's correlation analysis was performed separately for polar/orbital PFC and parietal lobe patients. Correlation of polar/orbital PFC patients’ accuracy scores in congruent neutral, congruent emotional, incongruent neutral, and incongruent emotional with volume loss in BA 10 and BA 11 did not show any significant results. The equivalent analysis for parietal lobe patients’ lesion volume loss in BA40 and BA 7 also did not result in any significant correlations.

## 4 Discussion

The current study investigated the effect of polar/orbital PFC and PL lesions on exclusive disjunctive reasoning with emotional and neutral content. The main result is a double dissociation between polar/orbital PFC and parietal lobes for emotional and neutral reasoning shown by the VLSM analyses. Patients with polar/orbital PFC lesions were impaired in the emotional content reasoning trials but not the neutral content reasoning trials. Patients with PL lesions displayed the reverse pattern. Normal controls provided a baseline for expected performance. Patients with PL lesions performed at the same level as normal controls in the emotional condition (but not in the neutral condition), and patients with lesions to polar/orbital PFC performed at the same level as normal controls in the neutral condition (but not in the emotional condition).

These results cannot be explained by group differences in age and education, nor by cognitive or emotional baseline scores, as the polar/orbital PFC and parietal lobe patients did not significantly differ on these measures from each other, or from normal controls. Other possibilities to account for these results might be group differences in believability of the argument conclusions (via the belief bias effect), or the emotional perception/impact of arguments. However, the data indicate no differences along these two dimensions.

A similar double dissociation was reported in a neuroimaging study involving categorical syllogisms with neutral content and negative emotional content (Goel and Dolan, 2003b). A content type (neutral, emotional) by task (reasoning, baseline) interaction revealed activation in medial ventral PFC for reasoning with negative emotional content, and activation in lateral/dorsal lateral PFC in reasoning with neutral content. The medial ventral PFC activation overlaps with the lesions displayed by our polar/orbital PFC patients, and these patients are indeed impaired in their ability to reason about emotional content, but not neutral content. The fact that patients with polar/orbital PFC lesions were unaffected in the neutral reasoning condition is consistent with many neuroimaging and patient studies that have failed to implicate this region in formal logical reasoning tasks (Baggio et al., 2016, Goel, 2007, Prado et al., 2011, Reverberi et al., 2012).

However, in the neutral condition, it is patients with lesions to the parietal lobes that are impaired. There are two possible reasons for this divergence in results between Goel and Dolan (2003b) and the present study. First, as noted above, our patient pool contained very few patients with lesions to left lateral PFC. Second, whether the parietal lobes or left PFC involved in reasoning with neutral content is a function of the logical forms of the argument and the presence (or absence) of meaningful content (Prado et al., 2011, Goel, 2007). In the case of categorical syllogisms with meaningful content that participants have beliefs about, it is a left lateral PFC and temporal system that is largely engaged (Prado et al., 2011, Goel, 2007).^3^ By contrast, transitive reasoning arguments engage the parietal lobes much more than the frontal lobes (Prado et al., 2011, Goel, 2007). There is little data about the neural systems underlying disjunctive reasoning. However, one study has reported stronger activation in left BA 40 in disjunctive reasoning than in conditional reasoning (Reverberi et al., 2007). This suggests a simple story of two separate reasoning mechanisms, one for emotional content, located in polar/orbital PFC, and one for neutral content, located in parietal lobes (at least for disjunctive arguments) that are selectively damaged in these patients.

However, when the results are further broken down to incorporate belief bias effects, a more complex story emerges. In articulating this story, we are assuming some form of a dual mechanism theory of logical reasoning (Evans, 2003). Dual mechanism accounts assume two separate systems for logical reasoning. There is a (fast, automatic) belief-based system for reasoning about familiar material that one has beliefs about. There is a second, (slow, effortful) formal reasoning system that is engaged when one is dealing with abstract, or unfamiliar material, that one does not have beliefs about. But most real-world reasoning involves familiar content. Whenever familiar content is involved, one will always encounter congruent and incongruent trials. In incongruent trials belief-based responses and logic-based responses can be differentiated. Responses based on believability of the conclusion lead to incorrect results. Correct responses are therefore indicative of (i) detection of a conflict between the believability of the conclusion and the validity of the argument, (ii) suppression of the prepotent belief-based response, and (iii) a formal evaluation of the argument. By contrast, in congruent trials belief-based responses and logic-based responses lead to the same correct conclusion, so are difficult to disentangle, though one might expect formal reasoning to lower accuracy.

One important result in the incongruent condition in the present study is a significant improvement of normal controls (and PL patients) in emotional content reasoning trials (Fig. 4C). Although it is commonly assumed that emotion hinders reasoning, some studies suggest that it can also be enhanced by negative emotional content (Blanchette et al., 2007, De Jong et al., 1997, Gangemi et al., 2013, Goel and Vartanian, 2011). Studies by Blanchette et al. (2007) and Goel and Vartanian (2011) have shown that normal healthy participants are overall more accurate on negative emotional incongruent trials compared to neutral incongruent trials in categorical syllogism tasks. One possible explanation for such results is that emotions may serve to attenuate belief bias effects.^4^ Negative emotionally charged sentences are not simply true or false, but are also subject to social approbation/disapprobation. This may warrant extra vigilance and recruitment of greater cognitive resources (attention and working memory allocation), which may improve reasoning performance by reducing reliance on belief bias, particularly in incongruent trials. In the case of the normal controls and parietal patients (with intact polar/orbital PFC), the emotional material triggers extra vigilance and allocation of greater cognitive resources, resulting in more systematic information processing.

The other important result from the analysis of the incongruent trials is the poor performance of the polar/orbital PFC patients in the emotional reasoning condition and their better performance in the neutral reasoning condition (compared to NC and PL patients) (Fig. 4C). One way of accounting for the former is to simply say that the polar/orbital PFC lesions preclude these patients from the above emotionally induced cognitive boost that normal controls and patients with parietal lobe lesions benefit from. Indeed, the performance of polar/orbital PFC patients on these emotional trials is very similar to the performance of NC and PL patients on the neutral incongruent trials. However, this does not explain how the polar/orbital PFC patients managed to outperform both, PL patients and normal controls in the neutral reasoning condition.

A second possibility is that lesions to polar/orbital PFC prevent patients from one or more of the following steps: detecting conflict, suppressing the prepotent response, and engaging in formal logical reasoning. But again, unless we assume that there are different mechanisms underlying these processes for neutral and emotional reasoning, it cannot explain the better performance of these patients in the neutral reasoning trials (requiring the same steps).

A third possibility is that polar/orbital PFC is a gating/purging mechanism for emotional content (Goel et al., 2017). On this account, formal reasoning occurs in lateral PFC and PL systems while conflict detection occurs in right dorsolateral PFC (Goel, 2007). Both of these systems are intact in the polar/orbital PFC patients (and NC). The input to these systems needs to be devoid of emotions. If emotional content leaks through they are unable to cope. This can explain poor performance of the polar/orbital PFC patients in the emotional reasoning condition, but again, it cannot explain improved performance of these patients in the neutral reasoning condition.

The most parsimonious explanation of the enhanced performance of patients with lesions to polar/orbital PFC in neutral incongruent trials may be that the lesions are attenuating belief bias effects in the neutral content reasoning condition. Goel and Dolan (2003b) showed that failure to detect a conflict/or suppress the prepotent response in (emotionally neutral) incongruent trials, results in a belief bias response which activates ventral medial PFC. It is possible that lesions to polar orbital PFC in our patients are resulting in blockage of the belief bias route in neutral reasoning trials, resulting in more frequent engagement of formal reasoning processes and increased accuracy in neutral incongruent trials.

This account also seems to explain the (nonsignificant) pattern of results in the congruent trials (Fig. 4B). The trend towards the polar/orbital PFC patients underperforming the normal controls in the neutral congruent trials can be explained with the same mechanism. If the belief bias route (which leads to the correct answer in this condition) is impaired, then the formal logic route must be used, leading to an elevated error rate.

So we are suggesting that lesions to polar/orbital PFC do two things: (i) prevent these patients from enjoying the emotionally induced cognitive boost (that NC and PL patients benefit from), and (ii) block the belief bias processing route in the neutral condition. There are data to support both of these roles for polar/orbital PFC in logical reasoning. For example, Goel and Dolan (2003b) found that emotional (“hot”) reasoning is associated with increased activation in the bilateral ventromedial prefrontal cortex in normal participants, and Goel and Dolan (2003a) reported that healthy volunteers activated bilateral vmPFC when giving belief-based responses in incongruent trials.

These results should be interpreted in the context of two caveats. First, as already noted, our patient population did not provide full brain lesion coverage. In particular, lesions to left lateral PFC were largely absent. Thus the study cannot speak to how lesions to these spared regions may have affected the results. Second, the modest number of stimuli utilized may have limited the detection power of the follow-up three way nonparametric analysis undertaken.

With respect to the performance of patients with parietal lobe lesions, overall, they do underperform in the neutral condition compared to the patients with polar orbital PFC lesions (Fig. 4A), but in comparison to the normal controls, they underperform (at a trend level) only in the congruent neutral condition (Fig. 4B). One possible explanation could be attenuation of belief bias effects, a finding in line with Baggio et al. (2016). This would result in more formal processing, and greater potential for errors. However, this explanation is inconsistent with their performance in neutral incongruent trials where they are as susceptible to belief bias effects as the normal controls. One possibility is that patients with lesions to PL are simply displaying a generalized impairment in neutral logical reasoning and it is failing to show up in the neutral incongruent condition due to a floor effect.

In summary, the current study shows that lesions to polar orbital PFC selectively impair reasoning on exclusive disjunctive items, with negative emotional content, compared to both, lesions to the parietal lobes and normal controls. Lesions to the parietal lobes selectively impair performance in neutral reasoning trials compared to lesions to polar orbital PFC. We are reluctant to accept a double dissociation account of the results due to the fact that the performance of patients with parietal lobe lesions does not statistically differ from that of normal controls. Upon breaking down the results in terms of belief bias effects, we find that patients with lesions to polar orbital prefrontal cortex underperformed in incongruent emotional content trials, and over performed in incongruent neutral content trials (compared to both normal controls and patients with parietal lobe lesions). Patients with lesions to parietal lobes underperformed (at a trend level) in congruent neutral trials.

We conclude that lesions to polar orbital PFC are having two effects. In the emotional content condition they are preventing these patients from benefiting from an emotion induced cognitive boost and, in the neutral condition, they are blocking the belief bias response route and forcing formal logical evaluation of arguments. Lesions to the parietal lobes are resulting in a generalized, nonspecific, impairment to logical reasoning, in the neutral condition.

The study replicates the findings of Goel et al. (2017), at least with respect to the impairment of polar orbital PFC patients in the emotional reasoning condition.^5^ This is important for two reasons. First, by performing a whole brain, VLSM analysis, rather than preselecting specific patients, we demonstrated that sensitivity to emotional content is specific to polar orbital PFC. Second, by using disjunctive reasoning items, we demonstrated that the effect is not limited to categorical syllogisms, and may be independent of logical form. The current results also diverge from Goel et al. (2017) in finding a significant improvement in the performance of polar orbital PFC patients in the incongruent neutral reasoning trials. This finding results in a modified explanation of the role polar orbital PFC in logical reasoning with emotional content. There is also a new finding of a significant overall impairment in the performance of patients with parietal lobe lesions in reasoning trials with neutral content.

## Footnotes

1 Not used in the VLSM analysis.

2 Although the main effects and interaction were not significant in the Congruent condition, visual inspection of graph Fig. 4B hints at possible between group differences in Neutral Content Congruent trials. Further investigation revealed borderline significance between polar/orbital PFC and Normal Controls (*U* = 116.00, *p* = .17, exact *p* = .016, *r* = .381 (Bonferroni corrected alpha < .017), a trend between Parietal and Normal Controls (*U* = 141.50, *p* = .060, exact *p* = .062, *r* = .298), and no significant difference between polar/orbital PFC and Parietal patients (*U* = 125.50, *p* = .676, exact *p* = .709, *r* = .073). There was no trend between group differences with Emotional Content trials (*p* ≥ .119).

3 In the absence of meaningful content, a bilateral frontal and parietal system is engaged (Goel, 2007).

4 It is also possible for emotional content to accentuate the belief bias effects, though there is no evidence of this in the present study.

5 Six participants in the current study matched experimental group participants in Goel et al. (2017) study (ID 0309, 0318, 1216, 1561, 2196, 3013). The correlation analysis between Emotional, Neutral, Emotional Congruent, Emotional Incongruent, Neutral Congruent, Neutral Incongruent accuracy scores between categorical syllogisms participants in Goel et al. (2017) and current data did not reveal any significant result (*r* ≤ .278, *p* ≥ .280).
