# Supplementary material for: Differential roles of polar orbital prefrontal cortex and parietal lobes in logical reasoning with neutral and negative emotional content
Source: Neuropsychologia. 2018 Oct;119:320–9. doi: 10.1016/j.neuropsychologia.2018.05.014 (PMC6200855; doi:10.1016/j.neuropsychologia.2018.05.014)
Supplement: Supplementary file 1 — Supplementary material [file mmc1.docx]

# Supplementary material:

**Emotional Disjunctions:**

1. Either there are pedophiles or politicians in Texas, but not both.

There are politicians in Texas.

There are no pedophiles in Texas. (valid/ implausible)

1. Either there are slaves or negroes in America, but not both.

There are no slaves in America.

There are no negroes in America. (invalid/implausible)

1. Either homosexuals are Christians or atheists, but not both

Homosexuals are atheists

Homosexuals may or may not be Christians (invalid/plausible)

1. Either in Korea there are edible dogs or opossums, but not both.

In Korea there are no opossums.

In Korea there are edible dogs. (valid/plausible)

### Neutral Disjunctions:

1. Either there are tigers or women in NYC, but not both.

There are no tigers in NYC.

There are women in NYC. (valid/plausible)

1. Either there are Christians or atheists in America, but not both.

There are no Christians in America.

There are no atheists in America. (invalid/implausible)

1. Either there exist pink elephants or white mice, but not both.

There are no white mice.

There are pink elephants. (valid/ implausible)

1. Either there is life on mars or Jupiter, but not both.

There is no life on mars.

There may or may not be life on Jupiter. (invalid/plausible)

Consider arguments #3 and #8. We included them to incorporate indeterminate (invalid) trials into the argument set (in addition to inconsistent trials). Technically these two arguments are valid, because if P follows from the premises, so does "P or not P". But the latter statement of the conclusion violates Gricean principles of "conversational logic" (Grice, 1975) and would be considered to be providing a "weaker" conclusion (in fact zero information, in terms of normal language use) than is warranted by the premises. We were looking for the most informative statement of the conclusion and therefore considered these two arguments as indeterminate (because they allowed for both possibilities). Such arguments were included and discussed in practice trials completed by participants.

To alleviate any concerns caused by this decision, we removed these trials and re-analysed the data with a non-parametric ANOVA, with within factors Content (Neutral, Emotional) and Congruency (Congruent, Incongruent), and between factor Group (polar/orbital PFC, PL, NC). The main effect of Group, Content and Congruency were significant (*ATS*(1.87) = 3.55, *p* = .031, *ATS*(1) = 28.10, *p* ≤ .001 and *ATS*(1) = 18.88, *p* ≤ .001, respectively) as well as Content by Congruency and Congruency by Group interactions (*ATS*(1) = 7.50, *p* = .006 and *ATS*(1.86) = 3.64, *p* = .030). The Group by Content and Group by Content by Congruency interactions were not significant (*ATS*(1.71) = 1.03, *p* = .348 and *ATS*(1.61) = 2.02, *p* = .142, respectively; Table S2).

Table S1: Mean (SD) accuracy for emotional and neutral content overall, congruent and incongruent trials for polar/orbital PFC, parietal lobe (PL) and normal controls (NC) samples

|  | Neutral | | | Emotional | | |
| --- | --- | --- | --- | --- | --- | --- |
|  | Congruent | Incongruent | Overall | Congruent | Incongruent | Overall |
| polar/orbital PFC | 0.64 (0.46) | 0.40 (0.45) | 0.52 (0.25) | 0.68 (0.37) | 0.43 (0.48) | 0.55 (0.30) |
| PL | 0.68 (0.43) | 0.24 (0.36) | 0.46 (0.20) | 0.85 (0.29) | 0.71 (0.47) | 0.78 (0.26) |
| NC | 0.91 (0.19) | 0.17 (0.29) | 0.54 (0.19) | 0.91 (0.19) | 0.76 (0.40) | 0.84 (0.25) |

Table S2: Lesion volume loss (cc) in prefrontal cortex in polar/orbital PFC group and Parietal Lobe (PL) patients

| Patient # | Right Hemisphere | | | | | | | | | Left Hemisphere | | | | | | | | | | | | |
| --- | --- | --- | --- | --- | --- | --- | --- | --- | --- | --- | --- | --- | --- | --- | --- | --- | --- | --- | --- | --- | --- | --- |
|  | BA10 | BA11 | BA47 | BA46 | BA7 | BA40 | BA39 | BA44 | BA45 | BA10 | | BA11 | BA47 | BA46 | BA7 | BA40 | | BA39 | BA44 | | BA45 |  |
| PL patients | | | | | | | | | | | | | | | | | | | | | |  |
| 230 | 0.0 | 0.0 | 0.0 | 0.0 | 0.2 | 0.0 | 0.0 | 0.0 | 0.0 | 0.0 | 0.0 | | 0.0 | 0.0 | 42.2 | | 4.4 | 0.3 | | 0.0 | 0.0 | |
| 408 | 0.0 | 0.0 | 0.0 | 0.0 | 26.1 | 0.1 | 0.0 | 0.0 | 0.0 | 0.0 | 0.0 | | 0.0 | 0.0 | 50.6 | | 1.2 | 14.7 | | 0.0 | 0.0 | |
| 439 | 0.0 | 0.0 | 0.0 | 0.0 | 37.9 | 25.9 | 59.3 | 0.0 | 0.0 | 0.0 | 0.0 | | 0.0 | 0.0 | 0.0 | | 0.0 | 0.0 | | 0.0 | 0.0 | |
| 1081 | 0.0 | 0.0 | 0.0 | 0.0 | 0.7 | 0.0 | 0.0 | 0.0 | 0.0 | 0.0 | 0.0 | | 0.0 | 0.0 | 0.0 | | 0.0 | 0.0 | | 0.0 | 0.0 | |
| 1149 | 0.0 | 0.0 | 0.0 | 0.0 | 0.0 | 23.0 | 21.5 | 0.0 | 0.0 | 0.0 | 0.0 | | 0.0 | 0.0 | 0.0 | | 0.0 | 0.0 | | 0.0 | 0.0 | |
| 1206 | 0.0 | 0.0 | 0.0 | 0.0 | 15.1 | 8.0 | 42.1 | 0.0 | 0.0 | 0.0 | 0.0 | | 0.0 | 0.0 | 0.0 | | 0.0 | 0.0 | | 0.0 | 0.0 | |
| 1298 | 0.0 | 0.0 | 0.0 | 0.0 | 7.7 | 56.7 | 0.7 | 0.0 | 0.0 | 0.0 | 0.0 | | 0.0 | 0.0 | 0.0 | | 0.0 | 0.0 | | 0.0 | 0.0 | |
| 1324 | 0.0 | 0.0 | 0.0 | 0.0 | 0.0 | 20.9 | 37.4 | 0.0 | 0.0 | 0.0 | 0.0 | | 0.0 | 0.0 | 0.0 | | 0.0 | 0.0 | | 0.0 | 0.0 | |
| 1366 | 0.0 | 0.0 | 0.0 | 0.0 | 30.5 | 0.0 | 14.3 | 0.0 | 0.0 | 0.0 | 0.0 | | 0.0 | 0.0 | 0.0 | | 0.0 | 0.0 | | 0.0 | 0.0 | |
| 1434 | 0.0 | 0.0 | 0.0 | 0.0 | 0.0 | 33.6 | 0.0 | 0.0 | 0.0 | 0.0 | 0.0 | | 0.0 | 0.0 | 0.0 | | 0.0 | 0.0 | | 0.0 | 0.0 | |
| 1443 | 0.0 | 0.0 | 0.0 | 0.0 | 17.0 | 12.5 | 39.4 | 0.0 | 0.0 | 0.0 | 0.0 | | 0.0 | 0.0 | 0.0 | | 0.0 | 0.0 | | 0.0 | 0.0 | |
| 1461 | 0.0 | 0.0 | 0.0 | 0.0 | 0.0 | 31.3 | 0.0 | 0.0 | 0.0 | 0.0 | 0.0 | | 0.0 | 0.0 | 0.0 | | 0.0 | 0.0 | | 0.0 | 0.0 | |
| 1519 | 0.0 | 0.0 | 0.0 | 0.0 | 5.4 | 0.9 | 0.0 | 0.0 | 0.0 | 0.0 | 0.0 | | 0.0 | 0.0 | 0.0 | | 0.0 | 0.0 | | 0.0 | 0.0 | |
| 1580 | 0.0 | 0.0 | 0.0 | 0.0 | 10.3 | 27.3 | 0.0 | 0.0 | 0.0 | 0.0 | 0.0 | | 0.0 | 0.0 | 0.4 | | 0.0 | 0.0 | | 0.0 | 0.0 | |
| 2028 | 0.0 | 0.0 | 0.0 | 0.0 | 29.5 | 33.5 | 28.9 | 0.0 | 0.0 | 0.0 | 0.0 | | 0.0 | 0.0 | 22.2 | | 32.2 | 0.0 | | 0.0 | 0.0 | |
| 2116 | 0.0 | 0.0 | 0.0 | 0.0 | 0.2 | 0.0 | 7.0 | 0.0 | 0.0 | 0.0 | 0.0 | | 0.0 | 0.0 | 0.0 | | 0.0 | 0.0 | | 0.0 | 0.0 | |
| 2182 | 0.0 | 0.0 | 0.0 | 0.0 | 0.7 | 19.1 | 0.0 | 0.0 | 0.0 | 0.0 | 0.0 | | 0.0 | 0.0 | 0.0 | | 0.0 | 0.0 | | 0.0 | 0.0 | |
| PFC patients | | | | | | | | | | | | | | | | | | | | | |  |
| 191 | 80.6 | 20.4 | 0.7 | 34.2 | 0.0 | 0.0 | 0.0 | 0.0 | 0.1 | 27.0 | 12.7 | | 0.0 | 0.0 | 0.0 | | 0.0 | 0.0 | | 0.0 | 0.0 | |
| 296 | 0.0 | 0.0 | 0.0 | 0.0 | 0.0 | 0.0 | 0.0 | 0.0 | 0.0 | 0.1 | 20.8 | | 38.5 | 0.0 | 0.0 | | 0.0 | 0.0 | | 0.0 | 0.0 | |
| 304 | 3.9 | 22.8 | 43.0 | 0.0 | 0.0 | 0.0 | 0.0 | 0.0 | 0.0 | 0.0 | 0.0 | | 0.0 | 0.0 | 0.0 | | 0.0 | 0.0 | | 0.0 | 0.0 | |
| 309 | 0.0 | 0.0 | 0.0 | 0.0 | 0.0 | 0.0 | 0.0 | 0.0 | 0.0 | 20.3 | 0.0 | | 0.0 | 9.1 | 0.0 | | 0.0 | 0.0 | | 0.0 | 0.0 | |
| 318 | 0.0 | 0.0 | 0.0 | 0.0 | 0.0 | 0.0 | 0.0 | 3.8 | 0.0 | 46.1 | 33.0 | | 10.6 | 0.0 | 0.0 | | 0.0 | 0.0 | | 0.0 | 0.0 | |
| 396 | 14.8 | 0.0 | 0.0 | 0.0 | 0.0 | 0.0 | 0.0 | 0.0 | 0.0 | 85.1 | 13.6 | | 6.5 | 7.9 | 0.0 | | 0.0 | 0.0 | | 0.0 | 7.6 | |
| 528 | 0.0 | 0.0 | 0.0 | 0.0 | 0.0 | 0.0 | 0.0 | 0.0 | 0.0 | 13.9 | 3.9 | | 6.9 | 9.3 | 0.0 | | 0.0 | 0.0 | | 4.7 | 18.6 | |
| 1127 | 17.5 | 16.4 | 0.1 | 0.0 | 0.0 | 0.0 | 0.0 | 0.0 | 0.0 | 9.4 | 8.2 | | 10.1 | 0.0 | 0.0 | | 0.0 | 0.0 | | 17.4 | 9.6 | |
| 1216 | 0.0 | 0.0 | 0.0 | 0.0 | 0.0 | 0.0 | 0.0 | 0.0 | 0.0 | 54.2 | 36.4 | | 0.0 | 3.0 | 0.0 | | 0.0 | 0.0 | | 0.0 | 0.0 | |
| 1407 | 2.2 | 52.5 | 1.8 | 0.0 | 0.0 | 0.0 | 0.0 | 0.0 | 0.0 | 0.0 | 0.2 | | 0.0 | 0.0 | 0.0 | | 0.0 | 0.0 | | 0.0 | 0.0 | |
| 1561 | 0.0 | 0.0 | 0.0 | 0.0 | 0.0 | 0.0 | 0.0 | 0.0 | 0.0 | 1.4 | 24.7 | | 10.0 | 1.2 | 0.0 | | 0.0 | 0.0 | | 0.0 | 4.3 | |
| 1662 | 24.8 | 56.6 | 1.9 | 0.0 | 0.0 | 0.0 | 0.0 | 0.0 | 0.0 | 76.5 | 70.1 | | 31.3 | 48.7 | 0.0 | | 0.0 | 0.0 | | 0.5 | 21.2 | |
| 1694 | 0.0 | 0.0 | 0.0 | 0.0 | 0.0 | 0.0 | 0.0 | 0.0 | 0.0 | 2.0 | 41.5 | | 55.2 | 0.0 | 0.0 | | 0.0 | 0.0 | | 0.0 | 1.8 | |
| 2196 | 26.1 | 9.7 | 0.0 | 0.3 | 0.0 | 0.0 | 0.0 | 0.0 | 0.0 | 0.0 | 0.0 | | 0.0 | 0.0 | 0.0 | | 0.0 | 0.0 | | 0.0 | 0.0 | |
| 2520 | 0.0 | 0.0 | 0.0 | 0.0 | 0.0 | 0.0 | 0.0 | 0.0 | 0.0 | 16.9 | 22.1 | | 0.6 | 0.0 | 0.0 | | 0.0 | 0.0 | | 0.0 | 0.0 | |
| 3013 | 46.2 | 21.4 | 0.0 | 2.2 | 0.0 | 0.0 | 0.0 | 0.0 | 0.0 | 0.0 | 0.0 | | 0.0 | 0.0 | 0.0 | | 0.0 | 0.0 | | 0.0 | 0.0 | |

Figure S1: *Mean accuracy rates (±1SEM) for (A) Exclusive Valid Disjunction items and (C) Exclusive Invalid Disjunction items as a function of Content (Emotional, Neutral) and group (polar/orbital (PFC), parietal lobe (PL), normal controls (NC)).*

*
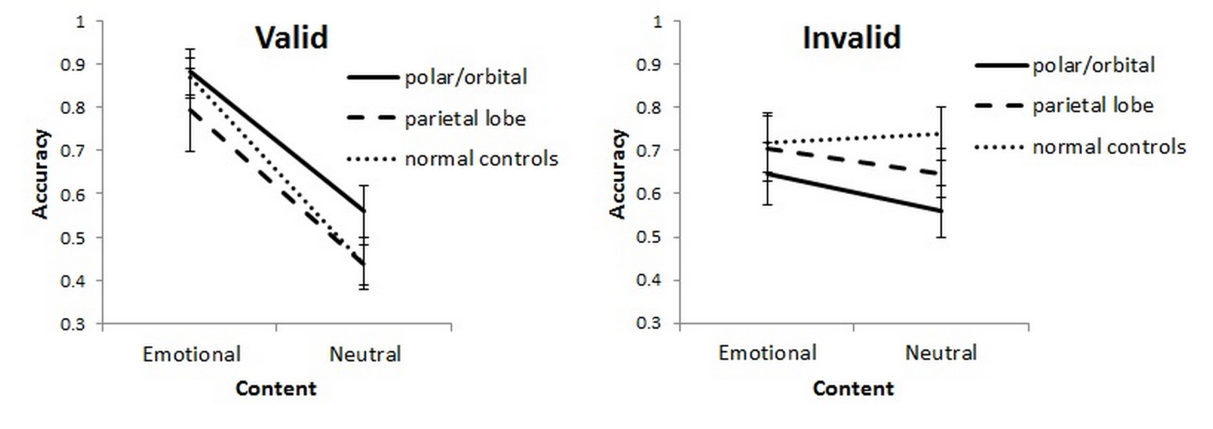
*
